# Does inflammation and altered metabolism impede efficacy of functional electrical stimulation in critically ill patients? Unleashing the potential of individualized functional electrical stimulation-cycling in critical illness

**DOI:** 10.1186/s13054-023-04788-w

**Published:** 2024-01-02

**Authors:** Murillo Frazão, Gerson Cipriano Jr., Paulo Eugênio Silva

**Affiliations:** 1 Lauro Wanderley University Hospital, Avenida Ruy Carneiro, 412, Miramar, João Pessoa, PB 58302-100 Brazil; 2 Health Sciences and Technologies Graduate Program, University of Brasilia (UnB), Brasilia, DF Brazil; 3 Graduate Program in Human Movement, Rehabilitation of Evangelical University of Goias, Anapolis, Brazil; 4 University of São Paulo Medical School, São Paulo, SP Brazil; 5 Hospital de Base Do Distrito Federal-IGESDF, Brasília, DF Brazil

Jameson et al. [1] recently published a study demonstrating intramuscular inflammation and altered substrate utilization in skeletal muscle in the first week of critical illness, with no effect following functional electrical stimulation-cycling (FES-cycling) intervention; these findings were also supported by two previous studies from the same group [2, 3]. Conversely, FES-cycling has been shown to promote a higher increase in cardiac output and peripheral oxygen extraction compared to other routine early mobilization methods used in critical illness [4], suggesting its potential for maintaining metabolic and physical function in these patients. The claimed ineffectiveness of FES-cycling by Jameson et al. [1] may be based on biased assumptions described in the subsequent.

According to the minimum standards required to ensure neuromuscular electrical stimulation clinical effects [5], we consider it crucial to indicate and discuss its concerns, providing a broader and updated view of this relevant rehabilitation intervention. In the earlier study [2], patients underwent FES-cycling with a 250 μs pulse width and a pulse amplitude varying from 0 to 60 mA, resulting in a total electrical charge [(pulse duration (μs) x pulse amplitude (mA)] ranging from 0 to 30,000 microcoulombs (μC) (Fig. 1). In the later study [3], patients underwent FES-cycling with a 250 μs (average-sized legs) or 300 μs (legs with edema) pulse width with a pulse amplitude varying from 20 to 30 mA, with a total electrical charge ranging from 10,000 to 18,000 μC (Fig. 1).

**Fig. 1:**
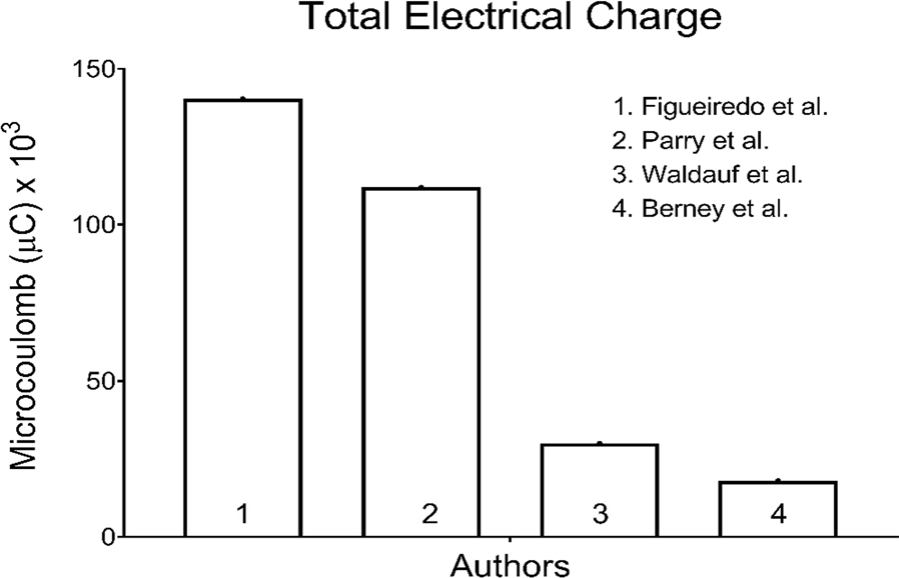
Average total electrical charge used in Figueiredo et al. [8] study and maximal total electrical charge used in Waldauf et al. [2] primary cohort, Berney et al. [3] validation cohort studies, and Parry et al. [9]

Critically ill patients commonly present neuromuscular electrophysiological disorders [6, 7], altering the neuromuscular excitability threshold, often resulting in a chronaxie ≥ 1000 μs. Figueiredo et al. [8] showed that critically ill patients have a high stimulation cost (i.e., the total electrical charge delivery rate per watt of output power). For optimal muscle performance, critically ill patients require an average total electrical charge of 140,400 μC (600 μs pulse width and 117 mA intensity) (Fig. 1), which is 4.7 times greater than the maximum used in the primary study [2] and 7.8 times than the maximum used in the validation cohort [3].

For optimal functional outcomes, precise parameter adjustments are also essential. Parry et al. [9] reported a 2.4 gain in physical function in intensive care test (PFIT) score in favor of the FES-cycling group, using a 300–400 μs pulse width and a maximum 140 mA of pulse amplitude (total electrical charge ranging from 84,000 to 112,000 μC) (Fig. 1). Meanwhile, the first study [2] achieved only a 1.3 PFIT score in favor of the FES-cycling group, and the validation study [3] reached a -0.2 PFIT score.

As the functional effects of FES-cycling in critically ill patients are dose-dependent, we advocate that individualized treatment based on neuromuscular excitability current characteristics (pulse width and pulse amplitude) is required. According to Maffiuletti et al. [5], the magnitude of electrically evoked force is the only valid indicator of neuromuscular electrical stimulation dose and the primary determinant of neuromuscular electrical stimulation treatment effectiveness. The literature presents evidence that, on average, pulse width should range from 500 to 1000 μs and pulse amplitude from 50 to 250 mA [8].

Finally, the number of stimulated muscles is a pivotal factor influencing neuromuscular electrical stimulation clinical and functional outcomes. Notably, in validation study [3], only one leg received FES-cycling. Volkers et al. [10] reviewed comparative studies of single versus double-leg active cycling. There is a higher hormonal levels of catecholamines as well as circulatory and ventilatory responses during double-leg cycling compared to one-leg cycling. Additionally, active muscle mass seems a crucial factor in the regulation of endurance performance. Consequently, exercise regimens that recruit a larger active muscle mass would optimally stress the release of biochemicals and hence the modulation of central training adaptations. It may positively affect physical capacity in, for example, persons that have diminished leg muscle mass available [10].

In conclusion, addressing neuromuscular excitability variations and optimizing intervention parameters, especially pulse width and amplitude, is crucial for personalized and effective rehabilitation. The need for further research persists to unlock the full potential of FES-cycling in enhancing outcomes for critically ill patients.

## Data Availability

Not applicable.
